# Evaluation of Yield and Drought Using Active and Passive Spectral Sensing Systems at the Reproductive Stage in Wheat

**DOI:** 10.3389/fpls.2017.00379

**Published:** 2017-03-29

**Authors:** Elisabeth Becker, Urs Schmidhalter

**Affiliations:** Department of Plant Science, Chair of Plant Nutrition, Technical University of MunichFreising, Germany

**Keywords:** carbon isotope discrimination, drought stress, ground cover, phenomics, precision phenotyping, spectral reflectance indices, water indices

## Abstract

Active and passive sensors are available for ground-based, high-throughput phenotyping in the field. However, these sensor systems have seldom been compared with respect to their determination of plant water status and water use efficiency related parameters under drought conditions. In this study, five passive and active reflectance sensors, including a hyperspectral passive sensor, an active flash sensor (AFS), the Crop Circle, and the GreenSeeker, were evaluated to assess drought-related destructive and non-destructive morphophysiological parameters (ground cover, relative leaf water content, leaf temperature, and carbon isotope discrimination of leaves and grain) and grain yield of twenty wheat (*Triticum aestivum* L.) cultivars. Measurements were conducted in a 2-year study, including a drought stress and a control environment under field conditions. A comparison of the active sensors at the heading, anthesis and grain-filling stages indicated that the Crop Circle provided the most significant and robust relationships with drought-related parameters (relative leaf water content and leaf and grain carbon isotope discrimination). In comparison with the passive sensor, the five water and normalized water indices (WI and NWI—1 to 4), which are only provided by the passive sensor, showed the strongest relationships with the drought stress-related parameters (*r* = −0.49 to −0.86) and grain yield (*r* = −0.88) at anthesis. This paper indicates that precision phenotyping allows the integration of water indices in breeding programs to rapidly and cost-effectively identify drought-tolerant genotypes. This is supported by the fact that grain yield and the water indices showed the same heritability under drought conditions.

## Introduction

Around the world, agriculture is challenged with an increased frequency of drought periods. An important issue is the reduction of available water for agricultural production, resulting in the stagnation and decrease of crop yields. Coincidentally, the global demand for agricultural products, especially corn, rice, and wheat, increases every year (Pingali, 2007; Tilman et al., 2011; Godfray, 2014). Wheat is one of the most extensively cultivated cereals that is often under abiotic stress (Cossani and Reynolds, 2012) and plays a crucial role regarding world food supplies (Shiferaw et al., 2013). Against this background, in a thirsty world, it is an absolute necessity to create drought-tolerant wheat phenotypes (Campos et al., 2006; Sinclair, 2011). Nonetheless, producing drought-tolerant wheat cultivars has proven complex under highly variable field conditions, and there is insufficient knowledge of physiological processes (Chaves et al., 2003; Campos et al., 2004; Boyer et al., 2013). Breeding new varieties for water-limited environments is still dominated by laborious field work and high priced laboratory analyses. In the last decades, a number of methods to evaluate drought stress have been established, such as the relative leaf water content (RLWC; Slatyer, 1967), leaf surface temperature (Blum et al., 1982; Reynolds et al., 1994), and carbon isotope discrimination (CID)(Farquhar et al., 1989; Condon et al., 2004). However, in large-scale field evaluations, these methods are expensive in terms of time and financial resources and partly require special equipment. Spectral canopy reflectance indices can also be used to assess plant water status because they change in response to crop water content (Penuelas et al., 1997; Stimson et al., 2005). Consequently, there is a great demand to increase breeding efficiency to guarantee the phenotyping of high numbers of lines in an exact and expeditious way (Araus and Cairns, 2014). In the last decades, numerous high-throughput phenotyping platforms (HTPPs) have been developed (Schmidhalter et al., 2001; Furbank and Tester, 2011) to accelerate the breeding process by screening various cultivars; these platforms offer detailed and non-invasive information about diverse plant parameters to determine plant water status (Schmidhalter, 2005; Winterhalter et al., 2011), leaf temperature (Rischbeck et al., 2014), and crop yield (Kipp et al., 2014a). These HTPPs carry either passive or active spectral sensors or a combination of both (Mistele and Schmidhalter, 2008, 2010; Erdle et al., 2011; Rischbeck et al., 2016), which can either be applied for scientific purposes or farm management. Passive sensor systems use sunlight as a source of light, whereas active sensor systems possess their own light-emitting units and therefore are independent of varying irradiation conditions and day and night (Hatfield et al., 2008). Furthermore, active sensors are frequently used due to their easy handling and relatively low purchase costs, which is especially attractive for developing countries. However, active sensors are limited to specific wavelengths according to the type of light source (Jasper et al., 2009; Erdle et al., 2011). Both sensor systems measure the reflection of a plant by converting the reflection signal into an electrical output. Hyperspectral passive sensors provide measurements of wavelengths in the visible (VIS; ~400–700 nm) and near-infrared (NIR; ~700–2,500 nm) ranges, which allows the calculation of different vegetation indices (Hackl et al., 2013). Therefore, spectral measurements from passive sensors can be applied to highly versatile conditions depending on the appropriate requirements (Hatfield et al., 2008; Erdle et al., 2011). Nonetheless, both sensor systems provide similar indices for estimating various plant parameters. One of the most widely used indices is the normalized difference vegetation index [NDVI = (R_780_−R_670_)/(R_780_+R_670_)]. The NDVI combines spectral information of the VIS and NIR regions and provides predictions of green biomass and photosynthetic capacity (Babar et al., 2006b). Furthermore, previous research has shown that wavelengths in the NIR region are appropriate to detect plant water status (Babar et al., 2006b; Gutierrez et al., 2010; Rischbeck et al., 2014; El-Hendawy et al., 2015). One of these NIR-based indices is the water index (WI = R_970_/R_900_), developed by Peñuelas et al. (1993). The WI has become an established index to detect RLWC under water-limited conditions. Based on the WI, Babar M. A. et al. (2006) developed two normalized water indices {NWI-1 = ([R_970_ − R_900_]/[R_970_ + R_900_]) and NWI-2 = ([R_970_ − R_850_]/[R_970_ + R_850_])} to screen spring wheat genotypes under drought conditions. In addition, Prasad et al. (2007) added the NWI-3 (NWI-3 = [R_970_ − R_880_]/[R_970_ + R_880_]) and NWI-4 (NWI-4 = [R_970_ − R_920_]/[R_970_ + R_920_]) for screening grain yield of winter wheat genotypes affected by drought stress. These five water indices (WI and NWI-1 to 4) demonstrated high potential for use as selection tools for grain yield in winter wheat under drought conditions (Prasad et al., 2007; El-Hendawy et al., 2015). One of the commercially available active sensors is the Crop Circle ACS-470® (Holland Scientific Inc., Lincoln, Nebraska), which is equipped with modulated polychromatic light emitting diodes (LEDs) as a source of light. The Crop Circle provides filters for 670, 730, and 760 nm to estimate the biomass and nitrogen status of various crops (Kipp et al., 2014b). In addition to the Crop Circle, the GreenSeeker (NTech Industries Inc., Ukiah, California) is also a widely used active sensor. The GreenSeeker includes two separate LEDs as sources of light and provides two fixed wavelengths at 774 nm and 656 nm to estimate green biomass and nitrogen supply in corn and wheat (Tremblay et al., 2009; Li et al., 2010; Shaver et al., 2010). In recent years, the high potential of active and passive sensors in estimating agronomic and physiological traits has been shown in various studies. Nevertheless, passive and active sensors have rarely been compared, and only little information is available regarding how diverse stressors, such as drought stress, influence the sensors' performance. The objectives of this study were therefore (1) to compare passive and active spectral sensor systems with respect to several indices and (2) to determine the potential of spectral indices to assess plant water status in a high-throughput mode by identifying the most reliable relationships with drought-related traits (leaf temperature, RLWC, CID) ground cover and yield under drought conditions.

## Materials and methods

### Experimental design, location details, and crop management

**Figure 1:**
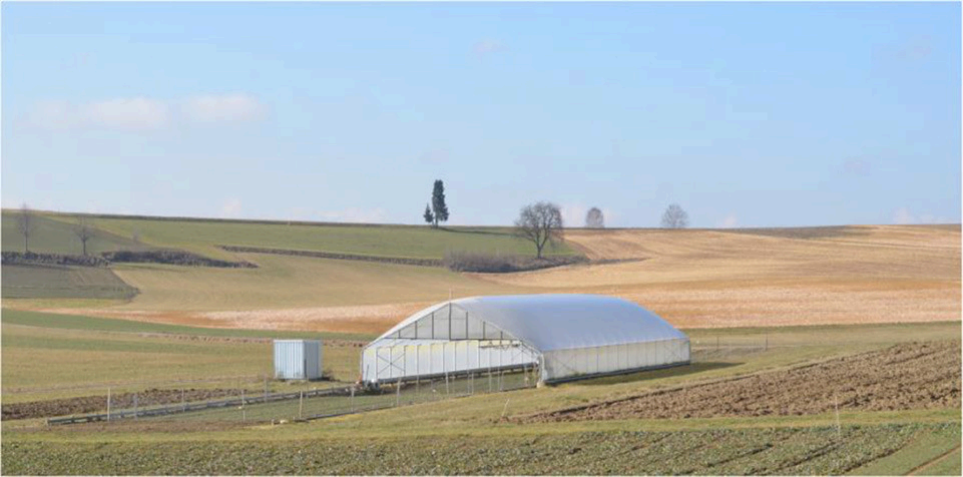
**Rain-out shelter at the research station Dürnast from the Technical University of Munich**.

The field study was conducted as a randomized block design consisting of four replicates arranged in six rows, in two seasons in 2014 and 2015 in a rain-out shelter (Figure 1) at the Dürnast research station of the Technical University of Munich in southern Germany (11°41′60″ E, 48°23′60″ N). Two different environments, one drought stress environment, created by withholding precipitation, and a control environment, grown next to the shelter with optimal water supply, were used to evaluate the drought tolerance of winter wheat (*Triticum aestivum* L.). Winter wheat plants were grown under natural weather conditions. In the case of rain, the shelter closed automatically and prevented water from reaching the plants. In this region, the average annual precipitation is ~800 mm with an average annual temperature of 8°C. The major demand for water by the crops occurs from April to the end of July; during this period, the average precipitation is ~350 mm with an average temperature of 13.7°C. The soil is characterized as a calcaric Cambisol consisting of silty loam. Twenty high-yielding wheat varieties (Supplementary Table 1) were grown in individual plots, consisting of eight rows spaced 15 cm apart with a length of 1.7 m. The sowing density was 350 kernels m^−2^. A total of 180 kg N ha^−1^ was applied as ammonium sulfate nitrate (ASS) at tillering (100 kg N ha^−1^) and as calcium ammonium nitrate (KAS) at stem elongation (80 kg N ha^−1^). All other nutrients, including P, K, S, and micronutrients, were supplied in adequate quantities to the plants. The plots were kept weed-free using integrated pest management.

### Spectral reflectance measurements

**Figure 2:**
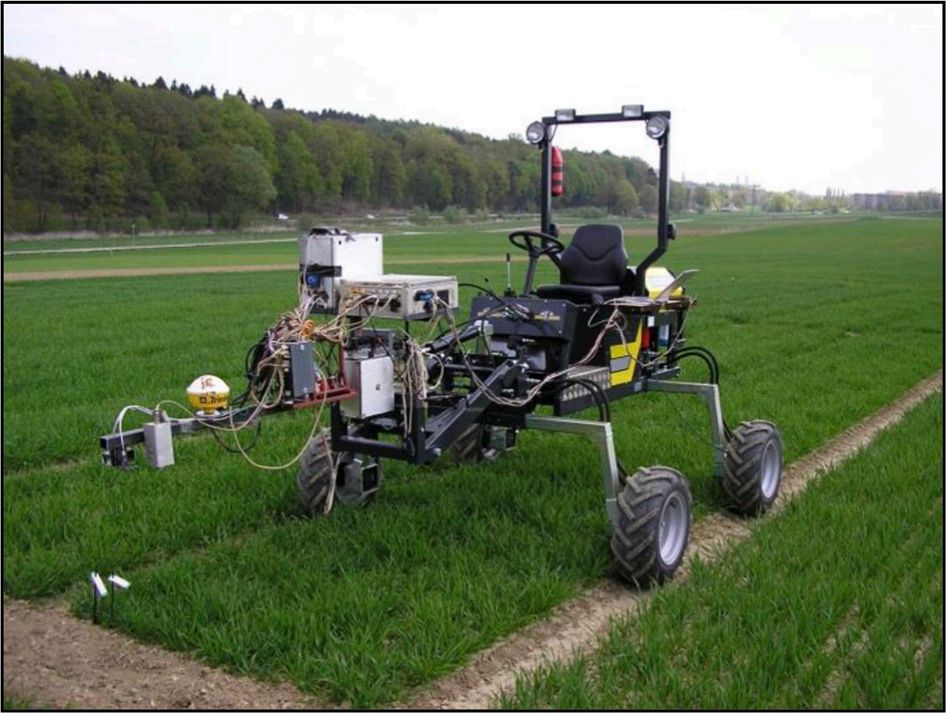
**PhenoTrac 4, carrying five passive and active spectral sensors**.

In parallel with RLWC, CID, and thermal measurements, spectral measurements were conducted using a passive spectrometer device enabling hyperspectral readings in a range of 400–1,200 nm and with a bandwidth of 3.3 nm (Mistele and Schmidhalter, 2010). The passive spectrometer included two Zeiss MMS1 silicon diode array spectrometers, which together measured canopy reflectance in a circular field of view (FOV) of ~0.28 m^2^ in the center of each plot. Measurements were recorded across the plot, covering ~¼ of the whole plot area. Additionally, solar radiation was detected as a reference signal with a second unit. In addition to the passive sensor, three active devices, a commercially available GreenSeeker RT100® (NTech Industries, Inc., Ukiah, CA, USA), a Crop Circle ACS-470® (670, 730 and 760 nm, Holland Scientific, Inc., Lincoln, NE) and an active flash sensor (AFS) similar to the N-Sensor ALS® (YARA International, ASA) but limited to a single sensor and a USB interface, were used. A light source flashing xenon light was included. This light source produced a spectral range of 650–1,100 nm with 10 flashes per second and a circular FOV of ~0.15 m^2^. The GreenSeeker included two LEDs, which detected the reflection in the VIS (656 nm, ~25-nm band width) and the NIR (774 nm, ~25-nm band width) spectral region. The FOV is a narrow strip with an approximate area of 0.009 m^2^ at a height of 66–112 cm above the plant canopy (NTech Industries, Inc., Ukiah, CA, USA, 2007). The Crop Circle operates in a similar way to the GreenSeeker. An advantage of the Crop Circle is that it provides more flexibility in the selection of detected wavelengths due to a choice of interference filters. For this study, filters for 670, 730, and 760 nm were selected. The FOV of the Crop Circle is an oval with an approximate area of 0.09 m^2^. For both active sensors, the FOV runs perpendicular to the sowing direction. The sensor device was mounted 1 m above the canopy in a nadir position on the mobile phenotyping platform PhenoTrac 4 developed by the Chair of Plant Nutrition, Technical University of Munich (http://www.pe.wzw.tum.de; Figure 2). Hence, simultaneous high-throughput measurements for all plots were obtained. Sensor readings were simultaneously recorded with GPS coordinates from a TRIMBLE RTK-GPS (real-time kinematic global positioning system; Trimble, Sunnyvale, CA, USA). In each plot, ~70 sensor readings were recorded and averaged. All measurements were conducted under cloudless sky at noon. To illustrate the different reflectance intensities in the VIS and NIR ranges of all used sensor systems, 10 indices were selected (Table 1). Because the active sensors are not always able to exactly detect the wavelengths of these indices, similar wavelengths and combinations were used to calculate ratios (Table 1) based on the six initial indices. In 2014, the active sensor Crop Circle was not available.

**Table 1:** **Indices and wavelengths of four sensor systems and the corresponding abbreviations**.

**Sensor**	**Index**	**Index abbreviation**
Passive	R_900_/R_970_	P_WI
	[R_970_−R_900_]/[R_970_+R_900_]	P_NWI-1
	[R_970_−R_850_]/[R_970_+R_850_]	P_NWI-2
	[R_970_−R_880_]/[R_970_+R_880_]	P_NWI-3
	[R_970_−R_920_]/[R_970_+R_920_]	P_NWI-4
	R_760_/R_670_	P_760/670
	R_774_/R_656_	P_774/656
	R_760_/R_730_	P_760/730
	R_730_/R_760_	P_730/760
	[R_780_−R_670_]/[R_780_+R_670_]	P_NDVI
Active flash sensor	R_900_/R_970_	ALS_WI
	R_760_/R_730_	ALS_760/730
	R_730_/R_760_	ALS_730/760
GreenSeeker	[R_774_−R_656_]/[R_774_+R_656_]	GS_NDVI
	R_7740_/R_656_	GS_774/656
Crop Circle	R_730_/R_670_	CC_730/670
	R_760_/R_730_	CC_760/730
	R_760_/R_670_	CC_760/670

### Leaf surface temperature

The leaf surface temperature was determined by thermometry. Two HEITRONICS KT15.83D infrared (IR) thermometers (Heitronics GmbH, Wiesbaden, Germany) were mounted opposed to each other on the PhenoTrac 4 at a 45° angle and with an FOV of 10 cm. The spectral range spanned 8–14 μm, and the temperature resolution was 0.06°C. All measurements were conducted in the center of each plot moving across the whole length. The temperature from both sensors was averaged to determine the leaf surface temperature.

### Relative leaf water content

At the heading, anthesis and grain-filling stages, the RLWC of the F-1 leaves was determined. Five leaves per plot were collected and immediately thereafter, the fresh weight (FW) was documented. To measure the turgid weight, the bottom part of the leaves was placed in sample tubes filled with distilled water for 16 h at 5°C in darkness. After 48 h at 60°C, the dry weight (DW) was measured. The RLWC was calculated according to the following formula:

(1) $$\begin{array}{r} RLWC\;\left(\%\right)=\frac{\left(FW-DW\right)}{\left(TW-DW\right)}\times 100 \end{array}$$

### Carbon isotope discrimination

The CID was determined using the F-1 leaves at the heading, anthesis, and grain-filling stages, as well as the grains at maturity. For each plot, five leaves were sampled and dried at 60°C for 48 h. At maturity, the grains of 15 plants were collected, ground to a fine powder and dried at 60°C for 48 h. The carbon isotope composition was measured using a mass spectrometer (Europe Scientific, Crewe, UK). The CID was calculated according to the following formula:

(2) $$\begin{array}{r} CID\left(‰\right)=\frac{\left(\delta a-\delta p\right)}{\left(1+\delta p\right)}x\;1000 \end{array}$$

where δa = δ^13^C of atmospheric CO_2_ (−8‰), and δp = δ^13^C of the sample (Farquhar et al., 1989)

### Ground cover measurements based on pixel analysis of RGB images

Images were captured using a Nikon D5100 reflex camera. To guarantee constant operational conditions, all images were captured under overcast conditions. The camera was manually held in a nadir position over the canopy at a height of 140 cm. In this position, approximately six rows of each plot were captured by the FOV of the camera. Digital image analyses of RGB images were conducted using ImageJ, a free, public domain Java image processing analysis program (Abràmoff et al., 2004). To differentiate green wheat pixels from brown soil pixels, thresholds for hue, saturation, and brightness were manually selected for each growth stage.

### Statistical analysis

SPSS 21 (SPSS Inc. Chicago, IL, USA) was used for statistical analysis. Simple linear regressions were calculated to analyze the relationship between different drought-related parameters and indices measured in this study. Correlation coefficients and significance levels were determined for nominal alpha values of 0.05, 0.01, 0.001, and 0.0001. Since lateral water influx affected the northern border row and two plots in the western heading column in 2014, this data was not considered for further evaluation.

### Calculation of heritability

#### Analysis within single treatments

Data were analyzed separately for each year. Within each treatment, data were analyzed using a linear model with the factors variety and replicate block. The significance of factors was determined using analysis of variance (ANOVA), and means were separated using Tukey's HSD test. The normality of distribution of the residuals was tested using the Shapiro-Wilk test. To calculate heritability, a model was fitted with both factors taken as random, using the lme4 package (Bates et al., 2014), and heritability on a mean basis was calculated as Vg/(Vg + Vr/r), where Vg and Vr are the genotypic and residual variance components, respectively, and r is the number of replicate blocks (Holland et al., 2003). All analyses were carried out using the R statistical package (R Core Team, 2016).

#### Analysis across treatments (within years)

To test for significant genotype–treatment interaction, a linear model with the factors variety, treatment, their interaction, and replicate block nested within treatments was fitted, and the significance was determined by ANOVA.

## Results

### Impact of drought stress on morphophysiological parameters

During both experimental years, and across the heading, anthesis and grain-filling stages, the drought-related parameters, i.e., RLWC, leaf temperature (LT), carbon isotope discrimination of leaves and grain (CIDL, CIDG), ground cover (GC), and grain yield, were measured (Table 2). The induced drought stress led to a statistically significant impairment of all morphophysiological parameters of the winter wheat plants during the three growth stages and in both experimental years. A significant decrease in RLWC, CIDL, CIDG, GC, and grain yield, as well as a significant increase in leaf temperature was observed compared with the control plants (Table 2).

**Table 2:** **Means [±standard error (SE)] of grain yield, carbon isotope discrimination (CID) of leaf and grain, leaf temperature (LT), relative leaf water content (RLWC), and ground cover (GC) at different growth stages during two experimental years**.

		**2014**				**2015**			
		**Drought**		**Control**		**Drought**		**Control**	
**Trait**	**GS**	**Mean**	**SE^b^**	**Mean**	**SE**	**Mean**	**SE**	**Mean**	**SE**
Grain yield [dt/ha]	Heading								
	Anthesis								
	Grain filling	86.08^a^	8.57	136.53^b^	8.32	99.83^a^	12.25	158.50^b^	11.34
RLWC [%]	Heading	76.69^a^	5.90	87.59^b^	4.14	79.02^a^	5.08	90.36^b^	3.15
	Anthesis	62.55^a^	3.79	84.02^b^	3.43	55.56^a^	3.29	90.22^b^	0.88
	Grain filling	45.16^a^	8.02	87.28^b^	2.72	40.33^a^	7.95	90.70^b^	2.20
LT [°C]	Heading	31.92^a^	2.45	27.50^b^	1.06	23.81^a^	2.48	21.82^b^	0.48
	Anthesis	27.76^a^	1.75	22.97^b^	0.81	32.88^a^	0.95	23.26^b^	0.36
	Grain filling	28.48^a^	1.93	21.99^b^	0.96	33.06^a^	1.84	21.75^b^	1.02
CIDL [‰]	Heading	19.96^a^	0.81	21.37^b^	0.42	21.09^a^	0.77	22.27^b^	0.19
	Anthesis	19.62^a^	0.51	22.64^b^	0.28	20.96^a^	0.36	22.21^b^	0.34
	Grain filling	19.08^a^	1.03	22.42^b^	0.43	19.10^a^	0.67	21.82^b^	0.41
CIDG [‰]	Heading								
	Anthesis								
	Grain filling	17.73^a^	0.53	20.59^b^	0.33	18.85^a^	0.62	21.03^b^	0.23
GC [%]	Heading	59.84^a^	17.48	92.44^b^	8.47	66.43^a^	6.07	91.14^b^	5.35
	Anthesis	55.18^a^	6.54	97.65^b^	0.84	60.68^a^	6.04	86.07^b^	5.09
	Grain filling	53.83^a^	13.45	87.70^b^	1.02	46.44^a^	13.06	65.97^b^	2.07

*Different superscripts show significant difference (Alpha = 0.05)*.

### Phenotypic correlation of drought-related parameters

Highly significant relationships were observed between all measured parameters for both experimental years and during the heading, anthesis and grain-filling stages (Table 3). All measured drought-related parameters exhibited strong phenotypic correlations (*r* > 0.50) with yield during all growth stages, but particularly at anthesis. The RLWC showed the weakest relationship with all other measured parameters. In the control environment, no obvious relationships were observed in either year or in any of the growth stages. A comparison of the heading, anthesis, and grain-filling stages of both experimental years indicated that measurements during anthesis were most closely related to grain yield (Table 3).

**Table 3:** **Correlations of drought−related parameters in winter wheat under drought and control conditions for heading, anthesis, grain filling (results of 2014 are presented in lower diagonal; results of 2015 are presented in the upper diagonal)**.

								**2015**					
		**RLWC**		**LT**		**CIDL**		**CIDG**		**GC**		**Yield**	
**Trait^a^**	**T^b^**	***r*****^c^**	**Sig.^d^**	***r***	**Sig**.	***r***	**Sig**.	***r***	**Sig**.	***r***	**Sig**.	***r***	**Sig**.
**HEADING**													
RLWC	DS			−0.41	^****^	−0.33	^***^	−0.38	^****^	0.38	^**^	**0.50**	^****^
	C			−0.03	ns	0.00	ns	−0.01	ns	0.11	ns	0.04	ns
LT	DS	−0.47	^****^			−**0.53**	^****^	−**0.60**	^****^	−**0.65**	^****^	−**0.75**	^****^
	C	**0.50**	^***^			−0.15	ns	−0.17	ns	−0.01	ns	0.04	ns
CIDL	DS	**0.52**	^****^	−**0.54**	^****^			**0.55**	^****^	**0.70**	^****^	**0.73**	^****^
	C	0.01	ns	−0.13	ns			0.20	ns	−0–17	ns	−0.15	ns
CIDG	DS	**0.65**	^****^	−**0.64**	^****^	**0.81**	^****^			**0.69**	^****^	**0.78**	^****^
	C	0.12	ns	−0.03	ns	0.26	ns			0−03	ns	0.20	ns
GC	DS	0.40	^***^	−0.40	^****^	0.31	^**^	0.47	^****^			**0.87**	^****^
	C	−0.07	ns	−0.02	ns	0.23	ns	0.02	ns			0.08	ns
yield	DS	**0.63**	^****^	−**0.67**	^****^	**0.74**	^****^	**0.79**	^****^	**0.51**	^****^		
	C	0.03	ns	−0.00	ns	−0.15	ns	0.21	ns	0.03	ns		
			2014										
**ANTHESIS**													
RLWC	DS			−0.44	^****^	0.47	^****^	**0.62**	^****^	**0.50**	^****^	**0.58**	^****^
	C			0.17	ns	−0.15	ns	0.06	ns	0.19	ns	0.06	^*^
LT	DS	−**0.65**	^****^			−**0.53**	^****^	−**0.60**	^****^	−**0.68**	^****^	−**0.74**	^****^
	C	0.11	ns			−0.24	^*^	−0.44	^***^	−0.23	^*^	−0.13	ns
CIDL	DS	**0.65**	^****^	−**0.74**	^****^			**0.56**	^****^	**0.70**	^****^	**0.74**	^****^
	C	0.08	ns	0.38	^*^			0.35	^**^	0.05	ns	0.06	ns
CIDG	DS	**0.57**	^****^	−**0.68**	^****^	**0.79**	^****^			**0.66**	^****^	**0.78**	^****^
	C	0.12	ns	0.03	ns	0.31	^*^			0.16	ns	0.20	ns
GC	DS	**0.54**	^****^	−**0.78**	^****^	**0.79**	^****^	**0.82**	^****^			**0.78**	^****^
	C	0.20	ns	0.14	ns	−0.08	ns	0.05	ns			0.20	ns
yield	DS	**0.59**	^****^	−**0.80**	^****^	**0.85**	^****^	**0.79**	^****^	**0.93**	^****^		
	C	0.16	ns	0.19	ns	0.09	ns	0.21	ns	0.39	^****^		
			2014										
**GRAIN FILLING**													
RLWC	DS			−0.47	^****^	0.41	^****^	**0.54**	^****^	0.48	^****^	0.45	^****^
	C			−0.06	ns	−0.16	ns	−0.06	ns	−0.19	ns	0.25	ns
LT	DS	−0.29	^**^			−**0.59**	^****^	−**0.60**	^****^	−**0.51**	^****^	−**0.66**	^****^
	C	−0.10	ns			0.34	^***^	−0.00	ns	−0.16	ns	−0.01	ns
CIDL	DS	0.33	^**^	−**0.67**	^****^			**0.57**	^****^	**0.54**	^****^	**0.70**	^****^
	C	−0.07	ns	−0.06	ns			0.25	^*^	−0.22	^*^	−0.05	ns
CIDG	DS	0.34	^**^	−**0.62**	^****^	**0.81**				**0.60**	^****^	**0.78**	^****^
	C	0.03	ns	−0.26	ns	−0.20	^****^			0.10	ns	0.20	ns
GC	DS	0.21	ns	−**0.72**	^****^	**0.69**	ns	**0.71**				**0.69**	^***^
	C	0.20	ns	0.09	ns	−0.13	ns	0.05	ns			0.14	ns
yield	DS	0.33	^**^	−**0.69**	^****^	**0.79**	^****^	**0.79**	^****^	0.86	^****^		
	C	0.03	ns	−0.00	ns	−0.15	ns	0.21	ns	0.59	^****^		
			2014										

a *RLWC relative leaf water content, LT leaf temperature, CIDL carbon isotope discrimination of leaf, CIDG carbon isotope discrimination of grain, GC ground cover, yield grain yield*.

b *Treatments, drought stress (DS), control (C)*.

c *r Correlation coefficient*.

d *Statistical significance as indicated by p−value ns non-significant*:

* *p < 0.05*,

** *p < 0.01*,

*** *p < 0.001*,

**** *p < 0.0001. Bold data display correlations > r = 0.50*.

### Phenotypic correlation of drought-related parameters and spectral indices

Selected indices from the VIS and NIR region, originating from the passive and active sensors, have been validated with respect to their ability to estimate drought-related parameters such as RLWC, LT, CIDL, CIDG, GC, and grain yield. At the heading and grain-filling stages, both sensor systems demonstrated similar capabilities with respect to estimating drought-related parameters. However, at anthesis, the passive sensors showed stronger relationships to the measured parameters compared with the active sensors. Furthermore, during anthesis and grain filling, the normalized water indices (NWI-1 to 4), which could only be calculated using the broad wavelength range of the passive sensor, demonstrated similar or stronger relationships to the drought-related parameters, GC and grain yield compared with the other indices (Table 4). Across all three growth stages and both experimental years, the active sensors showed a slightly stronger relationship to RLWC than the passive sensor. When comparing the heading, anthesis, and grain-filling stages for both experimental years, measurements during anthesis and grain filling provided the closest relationships (Table 4). Measurements conducted by the passive sensor tended to be more stable for all three growth stages, especially during anthesis.

**Table 4:** **Correlations of drought−related parameters, yield, and selected indices of passive and active sensors in winter wheat in drought and control environments for heading, anthesis, grain filling**.

		**RLWC**				**LT**				**CIDL**				**CIDG**				**GC**				**Yield**			
		^**′**^**14**		^**′**^**15**		^**′**^**14**		^**′**^**15**		^**′**^**14**		^**′**^**15**		^**′**^**14**		^**′**^**15**		^**′**^**14**		^**′**^**15**		^**′**^**14**		^**′**^**15**	
**Indices^a^**	**T^b^**	***r*^c^**	**Sig.^d^**	***r***	**Sig**.	***r***	***r***	**Sig**.	***r***	**Sig**.	**Sig**.	***r***	**Sig**.	***r***	**Sig**.	***r***	**Sig**.	***r***	**Sig**.	***r***	**Sig**.	***r***	**Sig**.	***r***	**Sig**.
**HEADING**																									
																								
P_R_760_/R_670_	DS	0.60	^****^	0.54	^****^	−0.55	^****^	−0.89	^****^	0.62	^****^	0.64	^****^	0.72	^****^	0.72	^****^	0.31	^*^	0.73	^****^	0.74	^****^	0.85	^****^
	C	−0.01	ns	−0.11	ns	0.05	ns	−0.02	ns	0.07	ns	0.13	ns	0.20	ns	0.11	ns	−0.10	ns	0.29	^**^	−0.12	ns	−0.05	ns
P_R_774_/R_656_	DS	0.43	^****^	0.48	^****^	−0.50	^****^	−0.77	^****^	0.48	^****^	0.66	^****^	0.56	^****^	0.61	^****^	0.17	^****^	0.78	^****^	0.70	^****^	0.84	^****^
	C	0.31	^*^	−0.11	ns	−0.26	ns	0.08	ns	−0.08	ns	0.04	ns	0.07	ns	0.16	ns	−0.10	ns	0.21	ns	−0.18	ns	0.10	ns
P_R_760_/R_730_	DS	0.33	^*^	0.30	^****^	−0.54	^****^	−0.15	^****^	0.46	^****^	0.38	^****^	0.50	^****^	0.23	^****^	0.24	^*^	0.50	^****^	0.64	^****^	0.47	^****^
	C	0.29	ns	−0.07	ns	−0.16	ns	−0.23	^*^	0.06	ns	0.06	ns	0.05	ns	0.11	ns	0.01	ns	0.13	ns	−0.12	ns	0.00	ns
P_R_730_/R_760_	DS	−0.62	^****^	0.55	^****^	0.64	^****^	−0.89	^****^	−0.71	^****^	0.64	^****^	−0.78	^****^	0.73	^****^	−0.48	^****^	−0.48	^****^	−0.85	^****^	0.84	^****^
	C	0.01	ns	−0.11	ns	−0.15	ns	0.06	ns	−0.04	ns	0.14	ns	−0.19	ns	0.09	ns	0.13	ns	0.31	^**^	0.20	ns	−0.07	ns
P_NDVI	DS	0.64	^****^	0.52	^****^	−0.60	^****^	−0.87	^****^	0.71	^****^	0.67	^****^	0.79	^****^	0.68	^****^	0.53	^****^	0.77	^****^	0.80	^****^	0.84	^****^
	C	−0.05	ns	−0.09	ns	0.11	ns	−0.04	ns	0.04	ns	0.10	ns	0.25	ns	0.10	ns	−0.11	ns	0.27	^**^	−0.17	ns	−0.08	ns
P_WI	DS	−0.37	^**^	−0.35	^****^	0.58	^****^	0.21	ns	−0.57	^****^	−0.42	^****^	−0.58	^****^	−0.33	^****^	−0.21	ns	−0.54	^****^	−0.65	^****^	−0.56	^****^
	C	0.27	ns	0.22	ns	0.19	ns	0.30	ns	0.14	ns	0.26	ns	−0.17	ns	−0.05	ns	0.12	ns	0.03	ns	0.17	ns	0.05	ns
P_NWI−1	DS	−0.37	^***^	−0.35	^**^	0.58	^****^	0.21	ns	−0.57	^****^	−0.43	^****^	−0.58	^****^	−0.33	^**^	−0.21	ns	−0.21	ns	−0.65	^****^	−0.57	^****^
	C	−0.27	ns	0.22	ns	0.18	ns	0.31	ns	0.14	ns	−0.26	ns	−0.16	ns	0.21	ns	0.12	ns	0.19	ns	0.17	ns	0.22	ns
P_NWI−2	DS	−0.39	^****^	−0.32	^**^	0.57	^****^	0.17	ns	−0.57	^****^	−0.41	^****^	−0.57	^****^	−0.29	^**^	−0.21	ns	−0.20	ns	−0.63	^****^	−0.51	^****^
	C	−0.26	ns	0.23	ns	0.14	ns	0.31	ns	0.20	ns	−0.30	ns	−0.17	ns	−0.22	ns	0.13	ns	0.21	ns	0.16	ns	0.23	ns
P_NWI−3	DS	−0.36	^***^	−0.35	^**^	0.58	^****^	0.22	ns	−0.56	^****^	−0.42	^****^	−0.57	^****^	−0.34	^**^	−0.21	ns	−0.21	ns	−0.66	^****^	−0.58	^****^
	C	−0.26	ns	0.23	ns	0.21	ns	0.30	ns	0.13	ns	−0.26	ns	−0.16	ns	−0.23	ns	0.11	ns	0.20	ns	0.17	ns	0.22	ns
P_NWI−4	DS	−0.37	^***^	−0.33	^**^	0.57	^****^	0.18	ns	−0.56	^****^	−0.41	^****^	−0.58	^****^	−0.30	^**^	−0.20	ns	−0.21	ns	−0.64	^****^	−0.54	^****^
	C	−0.27	ns	0.21	ns	0.16	ns	0.31	^*^	0.17	ns	−0.29	ns	−0.16	ns	−0.22	ns	0.11	ns	0.20	ns	0.17	ns	0.23	ns
ALS_WI	DS	0.12	ns	0.42	^****^	−0.24	^*^	−0.71	^****^	0.24	^*^	0.46	^*^	0.23	^**^	0.53		0.24	^*^	0.61	^****^	0.27	ns	0.63	^*^
	C	0.39	^*^	−0.12	ns	0.44	^**^	−0.11	ns	−0.07	ns	0.10	ns	0.08	ns	−0.02	ns	0.05	ns	0.23	^*^	−0.04	ns	−0.03	ns
ALS_R_760_/R_730_	DS	0.65	^****^	0.51	^****^	−0.62	^****^	−0.79	^****^	0.69	^****^	0.52	^****^	0.79	^****^	0.58		0.44	^****^	0.65	^****^	0.80	^****^	0.72	^****^
	C	−0.03	ns	−0.12	ns	0.10	ns	−0.10	ns	0.10	ns	0.20	ns	0.33	^*^	−0.03	ns	−0.12	ns	0.25	^*^	0.08	ns	−0.06	ns
ALS_R_730_/R_760_	DS	−0.65	^****^	−0.50	^****^	0.63	^****^	0.79	^****^	−0.70	^****^	−0.53	^****^	−0.80	^****^	−0.55		−0.48	^****^	−0.65	^****^	−0.79	^****^	−0.70	^****^
	C	0.03	ns	0.12	ns	−0.10	ns	0.11	ns	−0.09	ns	−0.20	ns	−0.32	^*^	0.03	ns	0.12	ns	0.25	^*^	−0.05	ns	0.06	ns
CC_R_760_/R_670_	DS	NA		0.58	^****^	NA		−0.82	^****^	NA		0.57	^****^	NA		0.70	^****^	NA		0.68	^****^	NA		0.80	^****^
	C	NA		−0.17	ns	NA		0.01	ns	NA		0.19	ns	NA		0.02	ns	NA		0.22	^*^	NA		−0.11	ns
CC_R_760_/R_730_	DS	NA		0.52	^****^	NA		−0.85	^****^	NA		0.56	^****^	NA		0.66	^****^	NA		0.67	^****^	NA		0.78	^****^
	C	NA		−0.12	ns	NA		−0.00	ns	NA		0.19	ns	NA		0.05	ns	NA		0.10	ns	NA		0.04	ns
CC_R_730_/R_760_	DS	NA		0.60	^****^	NA		−0.80	^****^	NA		0.58	^****^	NA		0.70	^****^	NA		0.68	^****^	NA		0.80	^****^
	C	NA		−0.17	ns	NA		0.02	ns	NA		0.17	ns	NA		0.01	ns	NA		0.25	^*^	NA		−0.16	ns
GS_NDVI	DS	0.62	^****^	0.59	^****^	−0.61	^****^	−0.79	^****^	0.57	^****^	0.57	^****^	0.69	^****^	0.65	^****^	0.42	^****^	0.70	^****^	0.70	^****^	0.77	^****^
	C	−0.07	ns	−0.02	ns	0.10	ns	−0.01	ns	0.16	ns	0.22	ns	0.20	ns	−0.02	ns	0.04	ns	0.18	ns	−0.12	ns	−0.09	ns
GS_R_774_/R_656_	DS	0.61	^****^	0.58	^****^	−0.58	^****^	−0.77	^****^	0.56	^****^	0.55	^****^	0.67	^****^	0.66	^****^	0.35	^*^	0.68	^****^	0.63	^****^	0.78	^****^
	C	−0.03	ns	−0.02	ns	0.08	ns	−0.01	ns	0.20	ns	0.23	^*^	0.20	ns	−0.02	ns	0.07	ns	0.18	ns	−0.08	ns	−0.09	ns
**ANTHESIS**																									
P_R_760_/R_670_	DS	0.55	^****^	0.47	^****^	−0.73	^****^	−0.77	^****^	0.70	^****^	0.65	^****^	0.79	^****^	0.61	^****^	0.83	^****^	0.83	^****^	0.51	^****^	0.84	^****^
	C	−0.27	ns	−0.18	ns	−0.29	^*^	−0.16	ns	0.10	ns	0.02	ns	0.23	ns	0.10	ns	−0.26	ns	0.14	ns	−0.16	ns	−0.16	ns
P_R_774_/R_656_	DS	0.51	^****^	0.42	^****^	−0.65	^****^	−0.74	^****^	0.63	^****^	0.62	^****^	0.71	^****^	0.61	^****^	0.75	^****^	0.77	^****^	0.76	^****^	0.80	^****^
	C	−0.19	ns	−0.11	ns	−0.43	^**^	−0.06	ns	0.10	ns	0.11	ns	0.24		−0.17	ns	−0.16	ns	0.08	ns	−0.09	ns	−0.10	ns
P_R_760_/R_730_	DS	0.54	^****^	0.45	^****^	−0.73	^****^	−0.64	^****^	0.71	^****^	0.64	^****^	0.76	^****^	0.52	^****^	0.84	^****^	0.81	^****^	0.84	^****^	0.82	^****^
	C	−0.22	ns	−0.15	ns	−0.38	^*^	−0.06	ns	0.10	ns	0.04	ns	0.15	ns	0.03	ns	−0.23	ns	0.17	ns	−0.19	ns	−0.27	^*^
P_R_730_/R_760_	DS	−0.53	^****^	0.44	^****^	0.77	^****^	−0.26	ns	−0.73	^****^	0.19	ns	−0.78	^****^	0.30	^*^	−0.87	^****^	0.16	ns	−0.85	^****^	0.20	ns
	C	0.24	ns	0.16	ns	0.30	^*^	0.14	ns	−0.12	ns	0.00	ns	−0.19	ns	−0.02	ns	0.22	s	−0.19	ns	0.20	ns	0.24	ns
P_NDVI	DS	0.54	^****^	0.42	^****^	−0.72	^****^	−0.74	^****^	0.69	^****^	0.63	^****^	0.76	^****^	0.59	^****^	0.82	^****^	0.81	^****^	0.81	^****^	0.82	^****^
	C	−0.25	ns	0.15	ns	−0.33	^*^	0.22	ns	0.10	ns	0.10	ns	0.26		0.10	ns	−0.21	ns	0.16	ns	0.13	ns	0.40	^**^
P_WI	DS	−0.60	^****^	−0.49	^****^	0.82	^****^	0.70	^****^	−0.80	^****^	−0.66	^****^	−0.82	^****^	−0.63	^****^	−0.85	^****^	0.81	^****^	−0.89	^****^	−0.86	^****^
	C	0.25	ns	0.10	ns	0.31	ns	0.10	ns	−0.11	ns	−0.13	ns	−0.28	ns	−0.22	ns	0.19	ns	−0.21	ns	−0.21	^**^	−0.37	^**^
P_NWI−1	DS	−0.60	^****^	−0.49	^****^	0.82	^****^	0.69	^****^	−0.80	^****^	−0.66	^****^	−0.82	^****^	−0.63	^****^	−0.85	^****^	−0.81	^****^	−0.89	^****^	−0.86	^****^
	C	0.25	ns	0.10	ns	0.31	ns	0.10	ns	−0.11	ns	−0.13	ns	−0.28	ns	−0.22	ns	0.19	ns	−0.21	ns	−0.21	^**^	−0.37	^**^
P_NWI−2	DS	−0.62	^****^	−0.50	^****^	0.80	^****^	0.74	^****^	−0.80	^****^	−0.66	^****^	−0.82	^****^	−0.65	^****^	−0.86	^****^	−0.82	^****^	−0.88	^****^	−0.88	^****^
	C	0.25	ns	0.10	ns	0.30	ns	0.11	ns	−0.14	ns	−0.11	ns	−0.28	ns	−0.21	ns	0.21	ns	−0.16	ns	−0.24	^**^	−0.36	^**^
P_NWI−3	DS	−0.60	^****^	−0.50	^****^	0.81	^****^	0.65	^****^	−0.81	^****^	−0.64	^****^	−0.82	^****^	−0.61	^****^	−0.85	^****^	−0.80	^****^	−0.90	^****^	−0.85	^****^
	C	0.26	ns	0.10	ns	0.30	ns	0.11	ns	−0.12	ns	−0.13	ns	−0.28	ns	−0.23	ns	0.20	ns	−0.21	ns	−0.23	^**^	−0.36	^**^
P_NWI−4	DS	−0.61	^****^	−0.49	^****^	0.81	^****^	0.68	^****^	−0.79	^****^	−0.64	^****^	−0.81	^****^	−0.62	^****^	−0.85	^****^	−0.82	^****^	−0.88	^****^	−0.87	^****^
	C	0.24	ns	0.09	ns	0.31	ns	0.10	ns	−0.14	ns	−0.12	ns	−0.28	ns	−0.21	ns	0.19	ns	−0.20	ns	−0.23	^**^	−0.37	^**^
ALS_WI	DS	0.53	^****^	−0.33	^*^	−0.50	^****^	−0.54	^****^	0.62	^****^	0.28	^*^	0.69	^****^	0.38		0.71	^****^	0.42	^****^	0.69	^****^	0.43	^****^
	C	−0.10	ns	−0.02	ns	−0.15	ns	−0.14	ns	0.14	ns	0.11	ns	0.34	^*^	−0.01	ns	0.22	ns	−0.18	ns	0.20	ns	0.15	ns
ALS_R_760_/R_730_	DS	0.56	^****^	0.32	^*^	−0.60	^****^	−0.70	^****^	0.63	^****^	0.51	^****^	0.71	^****^	0.55	^****^	0.81	^****^	0.70	^****^	0.71	^****^	0.73	^****^
	C	−0.23	ns	−0.06	ns	−0.16	ns	−0.04	ns	0.19	ns	0.18	ns	0.33	^*^	0.01	ns	−0.06	ns	−0.23	ns	0.08	ns	0.07	ns
ALS_R_730_/R_760_	DS	−0.57	^****^	0.52	^****^	−0.65	^****^	−0.22	ns	−0.66	^****^	−0.18	ns	−0.74	^****^	0.19	ns	−0.82	^****^	0.15	ns	−0.73	^****^	0.18	ns
	C	−0.24	ns	0.07	ns	−0.13	ns	−0.04	ns	−0.19	ns	−0.17	ns	−0.32	^*^	0.01	ns	0.08	ns	0.24	ns	−0.05	ns	0.07	ns
CC_R_760_/R_670_	DS	NA		0.52	^****^	NA		−0.78	^****^	NA		0.58	^****^	NA		0.56	^****^	NA		0.75	^****^	NA		0.75	^****^
	C	NA		0.01	ns	NA		−0.16	ns	NA		0.17	ns	NA		0.06	ns	NA		−0.18	ns	NA		0.23	ns
CC_R_760_/R_730_	DS	NA		0.49	^****^	NA		−0.76	^****^	NA		0.57	^****^	NA		0.59	^****^	NA		0.77	^****^	NA		0.78	^****^
	C	NA		−0.05	ns	NA		−0.20	ns	NA		0.09	ns	NA		0.10	ns	NA		−0.12	ns	NA		0.17	ns
CC_R_730_/R_760_	DS	NA		0.53	^****^	NA		−0.78	^****^	NA		0.58	^****^	NA		0.60	^****^	NA		0.74	^****^	NA		0.76	^****^
	C	NA		0.01	ns	NA		−0.12	ns	NA		0.17	ns	NA		0.05	ns	NA		−0.17	ns	NA		0.21	ns
GS_NDVI	DS	0.59	^****^	0.49	^****^	−0.64	^****^	−0.70	^****^	0.65	^****^	0.56	^****^	0.70	^****^	0.51	^****^	0.73	^****^	0.68	^****^	0.71	^****^	0.69	^****^
	C	0.29	ns	0.01	ns	−0.27	ns	0.02	ns	0.21	ns	0.06	ns	0.20	ns	0.01	ns	−0.29	ns	−0.23	ns	0.12	ns	0.27	^*^
GS_R_774_/R_656_	DS	0.58	^****^	0.52	^****^	−0.64	^****^	−0.71	^****^	0.63	^****^	0.55	^****^	0.69	^****^	0.51	^****^	0.70	^****^	0.67	^****^	0.72	^****^	0.68	^****^
	C	0.24	ns	−0.00	ns	−0.30	ns	−0.00	ns	0.23	ns	0.05	ns	0.20	ns	0.01	ns	−0.26	ns	−0.24	ns	0.08	ns	0.27	^*^
**GRAIN FILLING**																									
P_R_760_/R_670_	DS	0.33	^***^	0.34	^***^	−0.71	^****^	−0.65	^****^	0.75	^****^	0.61	^****^	0.78	^****^	0.61	^****^	0.45	^****^	0.50	^****^	0.81	^****^	0.84	^****^
P_R_774_/R_656_	DS	0.33	^***^	0.34	^**^	−0.71	^****^	−0.66	^****^	0.75	^****^	0.61	^****^	0.78	^****^	0.61	^****^	0.75	^****^	0.63	^****^	0.81	^****^	0.84	^****^
	C	−0.26	^*^	−0.25	^*^	−0.37	^*^	0.21	ns	0.18	ns	0.18	ns	0.23	ns	0.10	ns	−0.25	nsn	−0.20	ns	−0.16	ns	−0.05	ns
P_R_760_/R_730_	DS	0.36	^***^	0.34	^***^	−0.73	^****^	−0.67	^****^	0.76	^****^	0.59	^****^	0.77	^****^	0.58	^****^	0.79	^****^	0.63	^****^	0.85	^****^	0.81	^****^
	C	−0.23	^*^	−0.15	ns	−0.37	^*^	0.08	ns	0.21	Ns	0.17	ns	0.17	ns	0.11	ns	−0.23	ns	−0.08	ns	−0.17	ns	0.01	ns
P_R_730_/R_760_	DS	−0.34	^***^	−0.31	^***^	0.75	^****^	0.65	^****^	−0.76	^****^	−0.58	^****^	−0.78	^****^	−0.55	^****^	−0.81	^****^	−0.62	^****^	−0.85	^****^	−0.82	^****^
	C	0.24	^*^	−0.16	ns	0.33	^*^	0.08	ns	−0.21	ns	−0.17	ns	−0.19	ns	0.11	ns	0.22	ns	−0.10	ns	−0.19	ns	0.00	ns
P_NDVI	DS	0.27	^**^	0.35	^***^	−0.72	^****^	−0.66	^****^	0.76	^****^	0.58	^****^	0.79	^****^	0.62	^****^	0.77	^****^	0.47	^****^	0.80	^****^	0.84	^****^
	C	−0.28	^**^	−0.29	^**^	−0.22	^*^	0.21	^*^	0.16	ns	0.18	ns	0.25	ns	−0.02	ns	−0.24	ns	0.02	ns	−0.17	ns	−0.08	ns
P_WI	DS	−0.36	^**^	−0.38	^****^	0.76	^****^	0.60	^****^	−0.75	^****^	−0.61	^****^	−0.78	^****^	−0.63	^****^	0.47	^****^	−0.67	^****^	−0.84	^****^	−0.86	^****^
	C	0.22	ns	−0.01	ns	0.30	ns	−0.06	ns	−0.26	ns	−0.12	ns	−0.23	ns	0.01	ns	0.19	ns	0.02	ns	0.22	ns	−0.09	ns
P_NWI−1	DS	−0.36	^**^	−0.38	^**^	0.76	^****^	0.60	^****^	−0.74	^****^	−0.61	^****^	−0.78	^****^	−0.63	^****^	0.47	^****^	−0.69	^****^	−0.84	^****^	−0.86	^****^
	C	0.22	ns	−0.01	ns	0.31	ns	−0.06	ns	−0.26	ns	−0.11	ns	−0.22	ns	0.01	ns	0.19	ns	0.02	ns	0.22	ns	−0.10	ns
P_NWI−2	DS	−0.35	^**^	−0.37	^****^	0.75	^****^	0.64	^****^	−0.75	^****^	−0.61	^****^	−0.80	^****^	−0.65	^****^	0.46	^****^	−0.65	^****^	−0.82	^****^	−0.87	^****^
	C	0.23	ns	−0.05	ns	0.31	ns	−0.05	ns	−0.29	ns	−0.14	ns	−0.22	ns	0.02	ns	0.20	ns	0.03	ns	0.23	ns	−0.10	ns
P_NWI−3	DS	−0.37	^**^	−0.39	^****^	0.76	^****^	0.60	^****^	−0.74	^****^	−0.60	^****^	−0.78	^****^	−0.62	^****^	0.48	^****^	−0.70	^****^	−0.85	^****^	−0.86	^****^
	C	0.23	ns	−0.06	ns	0.30	ns	−0.08	ns	−0.26	ns	−0.13	ns	−0.23	ns	0.02	ns	0.18	ns	0.02	ns	0.22	ns	−0.10	ns
P_NWI−4	DS	−0.35	^**^	−0.38	^**^	0.76	^****^	0.62	^****^	−0.74	^****^	−0.61	^****^	−0.78	^****^	−0.64	^****^	0.45	^****^	−0.68	^****^	−0.83	^****^	−0.87	^****^
	C	0.21	ns	−0.06	ns	0.31	ns	−0.06	ns	−0.29	ns	−0.14	ns	−0.22	ns	0.02	ns	0.20	ns	0.02	ns	0.23	ns	−0.11	ns
ALS_WI	DS	0.34	^**^	0.33	^**^	−0.45	^****^	−0.57	^****^	0.60	^****^	0.48	^****^	0.68	^****^	0.54	^****^	0.59	^****^	0.36	^**^	0.69	^****^	0.64	^****^
	C	−0.11	ns	−0.09	ns	−0.14	ns	0.02	ns	0.13	ns	0.07	ns	0.34	^*^	−0.02	ns	0.20	ns	−0.07	ns	0.21	ns	−0.03	ns
ALS_R_760_/R_730_	DS	0.35	^***^	0.34	^***^	−0.62	^****^	−0.67	^****^	0.76	^****^	0.55	^****^	0.79	^****^	0.51	^****^	0.73	^****^	0.41	^****^	0.80	^****^	0.72	^****^
	C	−0.23	ns	−0.07	ns	−0.30	^***^	0.08	ns	0.20	ns	0.07	ns	0.33	^*^	−0.03	ns	−0.08	ns	−0.09	ns	0.08	ns	−0.06	ns
ALS_R_730_/R_760_	DS	−0.32	^**^	−0.31	^**^	0.63	^****^	0.65	^****^	0.76	^****^	−0.54	^****^	−0.80	^****^	−0.50	^****^	−0.74	^****^	−0.38	^****^	−0.80	^****^	−0.71	^****^
	C	0.24	ns	0.09	ns	0.25	^*^	−0.08	ns	−0.20	ns	−0.07	ns	−0.32	^*^	0.03	ns	0.10	ns	0.09	ns	−0.05	ns	0.06	ns
CC_R_760_/R_670_	DS	NA		0.48	^***^	NA		−0.74	^****^	NA		0.56	^****^	NA		0.70	^****^	NA		0.49	^****^	NA		0.72	^****^
	C	NA		−0.12	ns	NA		0.27	^*^	NA		0.12	ns	NA		0.02	ns	NA		−0.13	ns	NA		−0.11	ns
CC_R_760_/R_730_	DS	NA		0.39	^***^	NA		−0.71	^****^	NA		0.53	^****^	NA		0.66	^****^	NA		0.45	^****^	NA		0.78	^****^
	C	NA		−0.13	ns	NA		0.18	ns	NA		0.08	ns	NA		0.05	ns	NA		−0.11	ns	NA		0.04	ns
CC_R_730_/R_760_	DS	NA		0.48	^***^	NA		−0.74	^****^	NA		0.57	^****^	NA		0.70	^****^	NA		0.49	^****^	NA		0.71	^****^
	C	NA		−0.11	ns	NA		0.27	^*^	NA		0.11	ns	NA		0.01	ns	NA		−0.11	ns	NA		−0.16	ns
GS_NDVI	DS	0.29	^**^	0.38	^***^	−0.62	^****^	−0.73	^****^	0.67	^****^	0.55	^****^	0.69	^****^	0.65	^****^	0.69	^****^	0.47	^****^	0.70	^****^	0.77	^****^
	C	−0.29	ns	−0.01	ns	−0.32	^*^	0.09	ns	0.08	ns	0.02	ns	0.20	ns	−0.02	ns	−0.27	ns	0.03	ns	−0.12	ns	−0.09	ns
GS_R_774_/R_656_	DS	0.37	^**^	0.43	^***^	−0.59	^****^	−0.73	^****^	0.68	^****^	0.54	^****^	0.67	^****^	0.54	^****^	0.64	^****^	0.48	^****^	0.70	^****^	0.78	^****^
	C	−0.24	ns	−0.01	ns	−0.40	^*^	0.09	ns	0.07	ns	0.03	ns	0.20	ns	−0.02	ns	0.25	ns	0.03	ns	−0.08	ns	−0.09	ns

a *P, passive sensor; WI, water index; NWI, normalized water index; ALS, active flash light; CC, Crop Circle; GS, GreenSeeker*;

b *T, trait*;

c *correlation coefficient*;

d *statistical significance as indicated by p-value; ns, non-significant*:

* *p < 0.05*,

** *p < 0.01*,

*** *p < 0.001*,

**** p < 0.0001, NA, not ascertained, 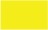
passive indices are stronger than active 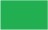
water indices are stronger than all other indices 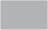
active indices are stronger than passive

### Heritability of drought-related parameters and spectral indices

In the drought environment, heritability for RLWC was moderate for both years (Table 5). During 2014, heritability was lower in the control compared with the drought environment. During 2015, under drought conditions, the genetic variance was estimated to be 0; hence, no heritability for RLWC could be calculated. Leaf temperature measurements, conducted by IR-Sensors, showed moderate heritability under drought conditions in 2014 and under control conditions in 2015. Moreover, the heritability of the CID was strong during 2014 for both environments and was moderate during 2015. Grain yield demonstrated a strong heritability under drought and control conditions for both experimental years. The studied water indices had moderate to strong heritabilities that were comparable with grain yield heritability under drought conditions during 2015 and 2014 (Table 5). The vegetation indices, determined by the passive sensor, demonstrated moderate heritabilities under drought conditions. Vegetation indices determined by the active sensors showed moderate heritabilities (ALS and GreenSeeker devices) and strong heritability (Crop Circle). For all active sensors, in most cases, the genetic correlation was estimated to be 0 in the control environment.

**Table 5:** **Heritability of drought-related parameters and spectral reflectance indices at anthesis under drought and control conditions**.

	**2014**		**2015**	
**Trait^a^**	**Drought**	**Control**	**Drought**	**Control**
	**h^2b^**	**h^2^**	**h^2^**	**h^2^**
RLWC	0.66	0.32	0.57	0
LT	0.52	0	0	0.42
CIDL	0.65	0.82	0.28	0.42
CIDG	0.72	0.86	0.44	0.39
GC	0.23	0	0	0.62
Yield	0.62	0.74	0.61	0.83
P_WI	0.53	0.24	0.60	0.74
P_NWI-1	0.52	0.24	0.61	0.74
P_NWI-2	0.54	0.19	0.46	0.80
P_NWI-3	0.54	0.35	0.49	0.75
P_NWI-4	0.54	0.13	0.43	0.78
P_760/670	0.41	0	0.45	0
P_774/656	0.34	0	0.31	0
P_760/730	0.22	0	0.49	0
P_730/760	0.19	0	0.18	0
P_NDVI	0.42	0	0.16	0.54
ALS_WI	0.78	0	0.14	0
ALS_760/730	0.45	0.48	0.41	0
ALS_730/760	0.35	0.48	0.67	0
GS_NDVI	0.58	0	0.11	0.54
GS_774/656	0.70	0	017	0.54
CC_730/670	NA	NA	0.75	0.23
CC_760/730	NA	NA	0.66	0.41
CC_760/670	NA	NA	0.61	0.11

a *RLWC, relative leaf water content (%); LT, leaf temperature FLIR-camera (C°); LT, leaf temperature IR-sensors (C°); CIDL, carbon isotope discrimination of leaf (‰); CIDG, carbon isotope discrimination of grain (‰); GC, ground cover (%); yield, grain yield (dt/ha); WI, water index; NWI1-4, normalized water indices; P, passive; ALS, active flash light; GS, GreenSeeker; CC, Crop Circle*.

b *Heritability*.

*NA, not ascertained*.

## Discussion

### Correlations between drought-related parameters

The assessment of plant water status provides information about the actual stress level under drought conditions. Measuring RLWC is a well-proven, direct indicator of the actual plant water status (Slatyer, 1967; Chaves et al., 2003). In the present study, a decrease in RLWC in response to increasing drought stress was observed during the heading, anthesis, and grain-filling stages (Table 2). Another approach for assessing plant water status is measuring leaf temperature. Measurements obtained using IR-sensors provide information on plant transpiration as the main contributor to reduce leaf temperature (Monneveux et al., 2012). This assumption was supported by significant negative correlations between RLWC and LT during all three growth stages (Table 3). Specifically, a low RLWC indicates a reduced transpiration rate as a water-saving strategy, which results in higher leaf temperatures. A lower transpiration rate leads to warmer leaves and lower stomatal conductance; both of these factors decrease net photosynthesis and crop duration (Monneveux et al., 2012). CID integrates stomatal conductance and photosynthesis capacity to transpiration over the life time of the organ being measured (Richards et al., 2010) and is considered as an indirect indicator of plant water status (Farquhar et al., 1989; Acevedo, 1993). For both experimental years, grain CID demonstrated strong linear relationships with grain yield; this finding agrees with the results reported from the studies conducted by Lopes and Reynolds (2010) and Araus et al. (2008). Moreover, leaf CID decreased with increasing drought stress (Table 3), agreeing with the results of Wang et al. (2016). At anthesis, leaf and grain CID exhibited strong positive relationships with RLWC and strong negative relationships with leaf temperature for both experimental years (Table 3). Thus, the assumption can be made that measurements of CID can be substituted with indirect measurements, such as leaf temperature measured using IR-sensors. This type of indirect measurement can be easily applied, is rapid and has a low cost. This is important since measurements of CID are associated with relatively high costs and the need for mass spectrometer facilities (Araus et al., 1997; Lobos et al., 2014). Furthermore, Monneveux et al. (2012) showed significant associations between leaf temperature and grain yield under drought conditions when measurements were conducted pre-anthesis and during grain filling. By contrast, our study demonstrated the strongest relationships with grain yield at anthesis for both experimental years (Table 3). Moreover, Monneveux et al. (2012) stated that under drought conditions, a relatively lower leaf temperature indicates a high capacity for taking up soil water to maintain a constant plant water status. During both experimental years, the ground cover showed strong relationships with the leaf temperature, RLWC and CID of the leaves and grains at anthesis (Table 3). Similar results were observed during grain filling, except for RLWC, which can be explained by a decrease in cell water due to progressive senescence. Briefly, in our study, low leaf temperatures, low CID and high RLWC were associated with higher ground cover. This leads to the supposition that more extensive ground cover helps to conserve soil moisture at the beginning of the growing season and is associated with relatively high net photosynthesis and cooler canopies. The digital ground cover approach offers several advantages over other measurement tools. To determine ground cover, no special equipment is needed, i.e., a commercial, affordable digital camera and free or inexpensive digitizing software (e.g., ImageJ: https://imagej.nih.gov/ij/) are sufficient. Among the three growth stages, the most significant and robust relationships were observed during anthesis, which represents the preferable growth stage to estimate drought stress. For this reason, heritability of all drought-related parameters was calculated at anthesis (Table 5). The drought stress parameter RLWC showed a moderate heritability (*h*^2^ = 0.57–0.66) in the drought environment. Furthermore, leaf and grain CID showed moderate to high heritability under drought stress (*h*^2^ = 0.28–0.72), which supports the observations of Rebetzke et al. (2008). The genetic variation of LT and GC in the control environment in 2014 and in the drought environment in 2015 was estimated to be 0; hence, heritability could not be calculated. As reported by Rebetzke et al. (2013), changes in cloud cover and wind speed and direction can potentially influence differences in leaf temperature among genotypes, which can negatively affect the calculation of heritability. Moreover, genotype × environment interactions and within-site variability contribute to larger sampling variance (Rebetzke et al., 2002; Richards et al., 2002). To our knowledge, this may the first study that provides a comprehensive comparison of a broad range of destructive and non-destructive morphophysiological parameters regarding their suitability to characterize drought stress under field conditions. In conclusion, measuring RLWC and CID of leaves and grains provided a good estimation of grain yield under drought stress at anthesis and also indicated high heritabilities. The main drawback of this approach is that the determination of both parameters is highly time consuming, error prone due to small sample sizes, and in the case of CID, associated with high financial expenses. By contrast, leaf temperature measurements using IR-sensors and the determination of ground cover provide an easy, low priced, and rapid measurement tool that is applicable to large field-scale experiments.

### Comparison of active and passive sensors with respect to the prediction of drought-related parameters and grain yield

In the last years, numerous studies have shown that different drought-related morphophysiological parameters can be measured and estimated simultaneously in a non-destructive and rapid way, providing that these parameters demonstrate a significant correlation with spectral information of the plant at different wavelengths under drought stress (Araus et al., 2001; Babar et al., 2006b; Erdle et al., 2011, 2013; Kipp et al., 2014a,b). For this purpose, several sensor systems are available, which are mainly classified as active and passive sensors. In the last decade, the potential of different active and passive sensor systems to assess agronomic and physiological parameters has been evaluated. However, the sensing principles have rarely been compared, and only limited information is available; therefore, further understanding is required. In this study, four reflectance sensors, including three active sensors (GreenSeeker, Crop Circle, ALS) and one passive, bi-directional hyperspectral sensor, which were all mounted on the mobile phenotyping platform PhenoTrac 4 (Figure 2), were tested under drought conditions and over 2 experimental years. All applied indices from all four sensor systems were significantly correlated with the morphophysiological parameters RWLC, LT, leaf, and grain CID, GC, and yield under drought stress (Table 4). At heading, all sensor systems, independent of the light source, provided comparable correlations with the measured parameters except for the drought stress parameter RLWC. RLWC is an adjuvant indicator of plant water status under drought stress (Slatyer, 1967; Chaves et al., 2003). The active sensors tended to yield slightly stronger relationships with RLWC compared with the passive sensor (Table 4). In addition, the vegetation indices R_760_/R_730_ and NDVI showed strong relationships with LT and GC for both experimental years. This fact indicates that these indices primarily detect the actual biomass, which was relatively high at heading due to moderate drought stress and was therefore associated with lower leaf temperatures and higher ground cover. Furthermore, the five NIR-based water indices (WI, NWI-1 to 4) showed similar relationships to the measured parameters, compared with the other applied indices, indicating that drought stress was not yet intensive enough to influence the plant reflectance in this range of wavelengths (800 ~ 900 nm). As a consequence of withholding precipitation, drought stress reached a severe level at anthesis (Table 2).

It is noticeable that at anthesis under severe drought stress, the passive sensor appeared to have an advantage over the active sensor systems, demonstrated by the stronger relationships with the measured parameters. This could be explained by differences in the sensor-dependent field of view (FOV). The passive sensor provides a larger FOV; thus, due to reduced ground cover of 55% (Table 2), the measured reflectance better reflected the actual drought conditions. Furthermore, it could be argued that the penetration depth of artificial light is lower compared with natural light, which is used by the active sensors. This assumption is supported by Jasper et al. (2009), Winterhalter et al. (2013), and Elsayed et al. (2015), who mentioned that the artificial light source of active sensors penetrates less deeply into crop canopies compared with solar radiation. An exception was observed for RWLC, as during the heading stage, the spectral estimation of this drought-related parameter was slightly better with the use of active sensors in both experimental years (Table 4), based on a comparison with the same indices. The first assumption was that RLWC is dependent on existing biomass and therefore, the active sensors could have had an advantage due to reduced spectral penetration, which is associated with reduced soil influence. However, this assumption could not be confirmed as the spectral indices of the passive sensor were more strongly related to GC than the active indices. This may be based on the fact that the measured plant reflectance of the active sensors mainly integrates the upper leaf levels, which represent the actual water status, especially under prolonged drought stress. The passive sensor includes spectral information on the whole plant, which could, due to increasing senescence, negatively influence the relationship with RLWC. Although the study of Bandyopadhyay et al. (2014) showed low correlations between RLWC and NWI-1 to 4, the five NIR-based water indices showed significant relationships with RLWC, which were on the same level as those observed for the active sensors. Moreover, the water indices exhibited highly significant relationships with LT and leaf and grain CID. Compared with the other applied indices, the water indices tended to have stronger relationships with most of the measured parameters. The NIR-based water indices compare the energy absorbed by water at 970 nm and different reference wavelengths of 850, 880, 900, and 920 nm, which do not indicate absorption by water (Penuelas et al., 1997; Prasad et al., 2007) and therefore are especially suited to detect plant water status.

The detection of leaf temperature is another indicator to quantify the drought stress level (Jones et al., 2009; Hackl et al., 2012; Rebetzke et al., 2013). In a study conducted by Babar et al. (2006a), positive relationships between canopy temperature and NIR-based indices at heading and grain filling were detected. Our findings reinforce these results and also show highly significant relationships between leaf temperature and the NIR-based indices at anthesis (Table 4). Furthermore, measurements of leaf and grain CID were conducted (Table 2). Measurements of CID are well-accepted as an indicator of water use efficiency (Araus et al., 2002; Blum, 2009). Only the study conducted by Lobos et al. (2014) reported on the relationship between CID and spectral indices for wheat during the middle of grain filling. In contrast to our findings, the study conducted by Lobos et al. (2014) showed no relationship between the NIR-based index and NWI-3. Further, our results indicate strong relationships between NWI-3 and CID for both leaves and grain under drought stress conditions for both experimental years (Table 4). In addition to the five water-based indices, the NDVI, which is associated with green biomass (Prasad et al., 2007), exhibited a good relationship with LT, leaf and grain CID and GC for both the active and passive sensors (Table 4). This indicates that green biomass contributes greatly to these relationships; however, the hypothesis is that that NIR at 970 nm penetrates deeper into the canopy, which probably estimates water content in a more precise way than other indices (Babar M. A. et al., 2006; Gutierrez et al., 2010). Therewith, the poorer relationships of the other indices from the active sensors as well as from the passive sensor could be explained. At grain filling, no explicit differentiation between the active and passive sensors could be observed (Table 4). The relationships between the evaluated indices (regardless of active or passive system) and RLWC were relatively weak, which is presumably associated with drought-induced premature senescence. By contrast, in 2015, the active sensors yielded a stronger relationship with RLWC compared with the passive sensor, albeit on a low relationship level. Furthermore, as already observed at anthesis, the five NIR-based indices tended to provide more robust spectral information compared with the selected indices. Briefly, in our study, the passive sensor yielded closer relationships with the measured destructive and non-destructive morphophysiological parameters compared with the active sensors. A comparison among the active sensors indicated that the Crop Circle yielded the most robust relationships. These findings support the results of Elsayed et al. (2015), who also made a comparison of different active sensors when measuring drought-stressed barley plants. In addition to the given indices of the active sensors and the equivalent indices and NIR-based indices of the passive sensor, a contour plot analysis, which tested all dual wavelength ratios of all measured parameters, was used to detect further suitable indices (data not shown). However, no combination of wavelengths could be detected that provided better estimations of the measured parameters than the applied water indices. However, as already shown by Erdle et al. (2011), the passive sensor proved to be more flexible to evaluate further indices due to the extended spectral range.

Grain yield represents the entire life of a plant and reflects the level of stress to which the plants have been exposed. In both experimental years, grain yield was reduced by ~60% due to the impact of drought stress (Table 2). During the three growth stages and both experimental years, highly significant relationships between spectral information and grain yield were detected, and the strongest correlations were observed at anthesis. However, during all growth stages, the indices of the passive sensor demonstrated up to ~20% stronger relationships with grain yield compared with the indices of the active sensors. Moreover, the five water indices (WI, NWI-1 to 4) consistently exhibited higher correlations with grain yield under drought conditions compared to the widely used indices (NDVI, R_760_/R_730_, R_730_/R_760_, etc.; see). These findings are consistent with the results of Prasad et al. (2007). The maximum correlation coefficient was observed at anthesis and grain filling for all five NIR-based indices, with a range from −0.85 to −0.90, indicating the efficiency of NIR-based indices for selecting drought-tolerant genotypes for grain yield production. The heritabilities of grain yield (*h*^2^ = 0.62) and the water indices (*h*^2^ = 0.52–0.61) were on the same level in the drought environment, over both experimental years, which supports the mentioned prediction. The heritability of the other applied active and passive indices ranged from 0.11 to 0.78 under drought stress; however, these indices did not provide estimates of the drought-related parameters and grain yield that were as reliable as those provided by the water indices. Prasad et al. (2007) reported that the water indices NWI-1 to 4 tended to explain more of the variability in grain yield when mean data, averaged over growth stages and years, were used. However, we succeeded in detecting highly significant relationships at individual growth stages, whereby all five water indices predicted grain yield under drought conditions. Indirect selection of secondary traits is a preferred selection approach when these traits have comparable heritability with the target traits (Gizaw et al., 2016). As reported by Jackson (2001), this applies especially when the secondary trait is easy to determine, is low priced, and is ascertainable in a high-throughput way. All of these requirements are fulfilled by the five NIR-based indices in our study. Furthermore, these indices demonstrated strong correlations with grain yield, and high heritabilities were observed for these indices. This could facilitate rapid measurements of a large number of plots used by breeders and farmer and could reduce the cost of individual measurements.

## Conclusions

Assessing plant water status RLWC and leaf and grain CID is associated with highly time-consuming measurements and costly analysis. In contrast, measurements of leaf temperature using IR-sensors and the determination of ground cover using digital cameras provide a rapid and easy approach to determine drought stress under field conditions, showing good relationships with grain yield and drought-related parameters. Moreover, at anthesis, spectral measurements using active or passive sensors demonstrated significant relationships with the measured destructive and non-destructive parameters, whereas the passive sensor tended to yield more robust estimations of the drought-related parameters. However, an exception to this was the parameter RLWC; the active sensors tended to yield a slightly stronger relationship with RLWC compared with the passive sensor. The NIR-based water indices (WI, NWI-1 to 4) demonstrated strong associations with the drought stress-related parameters (leaf temperature, RLWC, CID) and explained a high proportion of the variability in grain yield. Furthermore, in the current study, the NIR-based indices were proven to be useful for indirect selection for grain yield. This was indicated by the fact that they exhibited the same heritability. In addition, the active sensors systems were more flexible in terms of light and diurnal effects. However, the investigations of the present study indicate that to select drought-tolerant genotypes in a rapid and cost-effective manner, and therefore to accelerate breeding progress, future investigations will require broad-range spectral information to optimize the phenotyping of specific plant traits under drought conditions. The passive spectrometer provided the development of novel indices, which might be further transferred into active sensors.

## Author contributions

EB and US conceived and designed the experiments; EB performed the experiments; EB analyzed the data; EB and US wrote the paper.

## Funding

This research was funded by the DFG (German Research Foundation) funded project SCHM 1456/6-1.

### Conflict of interest statement

The authors declare that the research was conducted in the absence of any commercial or financial relationships that could be construed as a potential conflict of interest.
