# Supplementary material for: Evaluation of Yield and Drought Using Active and Passive Spectral Sensing Systems at the Reproductive Stage in Wheat
Source: Front Plant Sci. 2017 Mar 29;8:379. doi: 10.3389/fpls.2017.00379 (PMC5372809; doi:10.3389/fpls.2017.00379)
Supplement: Supplementary file 1 [file Table1.docx]

**Supplemental Table 1**: Overview of winter wheat cultivars

| Cultivar | Usage |
| --- | --- |
| Akteur | Bread wheat |
| Anapolis | Fodder wheat |
| Colonia | Bread wheat |
| Elixer | Biscuit, fodder, malting wheat |
| Genius | Bread wheat |
| Hybery | Bread wheat |
| Hybred | Bread wheat |
| Hyfi | Bread wheat |
| Hyland | Bread wheat |
| Hylux | Bread wheat |
| Hystar | Bread wheat |
| Impression | Bread wheat |
| JB Asano | Bread wheat |
| Kometus | Bread wheat |
| Manager | Bread wheat |
| Mulan | Bread wheat |
| Patras | Bread wheat |
| Piko | Hybrid father line |
| SUR.99820 | Hybrid mother line |
| Tobak | Bread wheat |
